# Cannabinoid compounds in combination with curcumin and piperine display an anti-tumorigenic effect against colon cancer cells

**DOI:** 10.3389/fphar.2023.1145666

**Published:** 2023-04-26

**Authors:** Büşra Yüksel, Ayşen Aslı Hızlı Deniz, Fikrettin Şahin, Kazim Sahin, Nezaket Türkel

**Affiliations:** ^1^ Department of Genetics and Bioengineering, Faculty of Engineering, Yeditepe University, Istanbul, Türkiye; ^2^ Department of Animal Nutrition, Faculty of Veterinary Medicine, Firat University, Elazig, Türkiye

**Keywords:** cannabidiol, cannabigerol, curcumin, piperine, colon cancer

## Abstract

Currently, use of cannabinoids is limited to improve adverse effects of chemotherapy and their palliative administration during treatment is curiously concomitant with improved prognosis and regressed progression in patients with different tumor types. Although, non-psychoactive cannabidiol (CBD) and cannabigerol (CBG) display antineoplastic effects by repressing tumor growth and angiogenesis both in cell line and animal models, their use as chemotherapeutic agents is awaiting further investigation. Both clinical and epidemiological evidence supported by experimental findings suggest that micronutrients such as curcumin and piperine may present a safer strategy in preventing tumorigenesis and its recurrence. Recent studies demonstrated that piperine potentiates curcumin’s inhibitory effect on tumor progression via enhancing its delivery and therapeutic activity. In this study, we investigated a plausible therapeutic synergism of a triple combination of CBD/CBG, curcumin, and piperine in the colon adenocarcinoma using HCT116 and HT29 cell lines. Potential synergistic effects of various combinations including these compounds were tested by measuring cancer cell proliferation and apoptosis. Our findings revealed that different genetic backgrounds of HCT116 and HT29 cell lines resulted in divergent responses to the combination treatments. Triple treatment showed synergism in terms of exhibiting anti-tumorigenic effects by activating the Hippo YAP signaling pathway in the HCT116 cell line.

## 1 Introduction

Colorectal cancer (CRC), which is a heterogeneous disease, involves the uncontrolled growth of cells in the rectum, colon, or gastrointestinal tract appendix (Fleming et al., 2012). Mostly because CRC patients receive their diagnosis at the later stages of the disease, CRC accounts for one of the highest mortality rate, corresponding to 883,200 deaths worldwide (WCRF/AICR, 2018). In addition to a higher mortality rate than other types of cancer, with 1.84 million cases recorded in 2018, CRC ranked as the second most commonly diagnosed cancer in females and the third in males (Bray et al., 2018). According to the stage of cancer and the degree of the complication, current treatment methods rely on the success of chemotherapy either alone or combined with surgical resection or radiation therapy. Although therapy options remain limited, there are life style factors known to reduce the risk of CRC. For example, maintaining proper dietary habits is an important component of promoting disease prevention. A recent study reported a lower incidence of CRC associated with vegetarian diets when compared to carnivore diets underscoring the importance of the diet as a risk factor in the occurrence of the disease (Orlich et al., 2015). Another epidemiological study revealed that it is possible to reduce CRC death rate by as much as 90% through inclusion of naturally existing bio-compounds with the anti-cancer and anti-oxidant characteristics such as curcumin that is shown to exert distinctive anti-tumorigenic properties in various models (Goel et al., 2001).

Curcumin, chemically known as diferuloylmethane, is a hydrophobic polyphenol naturally present in the rhizome of the plant *Curcuma longa* (turmeric) (Nelson et al., 2017). It is suggested that curcumin can selectively kill tumor cells through its multifaceted metabolic effects, that culminate in its anti-oxidant and anti-inflammatory activities (Hewlings and Kalman, 2017). Several clinical trials classify curcumin as a potential chemo-preventive and chemotherapeutic agent (Doello et al., 2018).

Mechanistically, numerous factors operating in several signaling pathways are implicated in mediating the anti-oxidant effect of curcumin such as induction of the Nuclear factor erythroid 2-related factor 2 (Nrf2) as a protective response against oxidative damage induced by ferric nitrilotriacetate (Fe-NTA) (BALOGUN et al., 2003). While its anti-inflammatory action involves inhibtion cyclooxygenase-2 (COX-2), lipoxygenase (LOX), inducible nitric oxide synthase (iNOS), and downregulation of Janus kinase (JAK) signal transducer and activator of transcription signaling pathways, all of which are essential for inflammatory processes (Shanmugam et al., 2015). In exerting its anti-tumorigenic effects, curcumin blocks angiogenesis, and negatively regulates cancer cell cycle progression as well as metastatic activity (Bhandarkar and Arbiser, 2007).

In several combinatorial therapy approaches, where a secondary active drug agent or drug candidate is co-administered with curcumin, an increase in the therapeutic benefit from curcumin has been reported in diverse cancer models (Baldi et al., 1839), (Bolat et al., 2020), (Schmidt et al., 2020). Strikingly, the second agent turns out to enhance curcumin-dependent anti-cancer activity in a synergistic fashion in certain cases.

Among the proposed secondary agents, piperine, a dietary polyphenol isolated from black and long peppers, distinguished with its intrinsic features, improves -not only-curcumin’s existing anti-cancer activity, but also its extremely poor bioavailability (Tang et al., 2017) (Tang et al., 2017) As a single agent, piperine alone also displays anti-mutagenic and anti-tumor activities (Chinta et al., 2015). For example, this agent can inhibit the proliferation of colon cancer cell lines via induction of a cell cycle arrest in the G1 phase, while it triggers apoptosis in prostate cancer models (Ouyang et al., 2013), (Yaffe et al., 2015).

Similar to curcumin and piperine, cannabinoids constitute another group of compounds that have been discovered as novel agents offering a promising anti-tumorigenic potential in multiple cancer types during their clinical use as palliative agents (Borrelli et al., 2014). Originally, cannabinoids were used to ameliorate the debilitating side effects of cytotoxic anti-cancer drugs such as anorexia, pain and emesis for a long time. Among more than 60 variants of cannabinoids, *Cannabis sativa*, and THC (delta-9-tetrahydrocannabinol), cannabidiol (CBD), cannabinol (CBN), cannabichromene (CBC), and cannabigerol (CBG), have been heavily studied (Sreevalsan et al., 2011; McAllister et al., 2015). For example, CBD inhibits the progression of many types of cancer, including glioblastoma multiforme (GBM), breast, lung, prostate and colon cancer (Orrego-González et al., 2020). Likewise, Cannabigerol (CBG) promotes apoptosis, stimulates reactive oxygen species production, and reduces cell growth in CRC cells. Moreover, CBG inhibits the progression of the chemically induced colon carcinogenesis and xenograft tumors *in vivo* (Borrelli et al., 2014). Remarkably, *Cannabis*-derived compounds display reduced cytotoxic behavior on normal colon cells, despite their well-established cytotoxic activity on colon carcinoma cells (Ben-Ami Shor et al., 2022).

Therefore, we chose CBD and CBG to pursue enhancing their demonstrated therapeutic potential in colon carcinoma through their supplementation with curcumin and piperine. Using human HT29 and HCT116 colon cancer cell lines, we made the first attempt to uncover the efficacy of a triple combination, where cannabinoid compounds CBD or CBG are added to a curcumin plus piperine dual cocktail (curcumin/piperine), both of which are considered inextricable in terms of providing most optimal therapeutic outcome possible. Our findings indicate that in the triple combinatory approach, these natural compounds exhibited enhanced anti-carcinogenic effects in colon cancer cells by inducing apoptosis and blocking cell proliferation. Finally, we demonstrate that the improved therapeutic potential of the triple combination entails the activation of the Hippo YAP signaling pathway.

## 2 Materials and methods

### 2.1 Cell lines and cell culture conditions

HT-29 (HTB-38, human colorectal adenocarcinoma cell lines), HCT-116 (CRL-247, human colorectal adenocarcinoma cell lines) were originally purchased from American Type Culture Collection (ATCC, Rockville, MD). HT-29 cell lines were cultured in Roswell Park Memorial Institute medium (RPMI, #11875093, Invitrogen, Gibco, UK). The HCT-116 cell line was cultured in Dulbecco’s Modified Eagle’s Medium (DMEM, #41966-029, Invitrogen, Gibco, UK). Each medium was supplemented with 1% Penicillin/Streptomycin/Amphotericin (PSA, Invitrogen, Gibco, UK) and 10% fetal bovine serum (FBS, #10500-064, Invitrogen, Gibco, UK). Cells were maintained at 37°C and 5% CO2 in a humidified incubator.

### 2.2 Cytotoxicity assay

Effects of curcumin (Sigma-Aldrich, Germany), piperine (Sigma-Aldrich, Germany), cannabidiol (CBD), cannabigerol (CBG) on cell viability of HCT-116 and HT-29 cells were tested. Stock solutions of 1 µM curcumin, 1 µM piperine, 500 μg/ml CBD, and 500 μg/ml CBG molecules were dissolved in DMEM (for HCT116) or RPMI (for HT29) containing DMSO in 1:100 ratio.

HCT116 and HT29 cells were cultured in 96-well plates at a density of 5,000 cells/well. The following day, cells were treated with curcumin (doses ranging from 100 µM to 10 µM), piperine (doses ranging from 80 µM to 1 µM), CBD (doses ranging from 100 μg/ml to 10 μg/ml), CBG (doses ranging from 100 μg/ml to 10 μg/ml), and curcumin piperine CBD combinations (doses ranging from 50 µM/10 µM/15 μg/ml to 10 µM/2 µM/15 μg/ml) curcumin piperine CBG combinations (doses ranging from 50 µM/10 µM/25 μg/ml to 10 µM/2 µM/25 μg/ml. After administering the cells with different concentrations of the compounds for 72 h as described in similar studies (Bolat et al., 2020), cell viability was assessed via MTS assay 3-(4,5-dimethyl-thiazol-2)-5-(3-carboxy-methoxy-phenyl)-2-(4-sulfo-phenyl)-2H-tetrazolim salt (MTS) (#G3582, CellTiter96 AqueousOne Solution; Promega, Southampton, UK) following the procedure used in the same study (Bolat et al., 2020). Treatment containing medium was removed, and an MTS solution (PBS solution included 10% MTS and 4.5 g/L d-glucose solution) was added followed by 90 min of incubation at 37°C. Then, their absorbance was measured at 490 nm by using an ELISA plate reader (Biotek, Winooski, VT). IC50 values were calculated by the GraphPad prism software.

### 2.3 Annexin V assay

Determination of IC50 values was followed by the investigation of these concentrations of the compounds on apoptosis by Annexin V assay. HCT-116 and HT-29 cells were seeded into T25 flasks at a density of 50 × 103. The following day, media was aspirated, and cells were treated with CBD (25 μg/ml for HT-29 and 15 μg/ml for HCT116), CBG 50 μg/ml for HT-29 and 25 μg/ml for HCT116), curcumin (25 µM for both cell lines), piperine (5 µM for both cell lines) and their combinations. After 72 h of treatment cells, Annexin V assay was performed according to manufacturer’s protocol (#sc-4252AK, Santacruz Biotechnology, United States). Cells were harvested and washed with ice-cold PBS. Then they were resuspended in Annexin V binding buffer and separated into four groups (Annexin V, propidium iodide (PI), Annexin V + PI, and NC). Cells were incubated for 15 min at room temperature for annexin V and PI staining (Kamiloglu et al., 2020) Data were analyzed by using FACSCalibur (BD biosciences) flow cytometry.

### 2.4 Cell cycle analysis

Cells were seeded into T25 flasks at a density of 50 × 103. The following day, treatments were applied, and cells were further incubated for 72 h at 37°C. Then, they were harvested and washed with PBS and fixed with 70% ice-cold ethanol for at least 2 hours at -20°C (Kim and Sederstrom, 2015). Cell pellets were permeabilized with 0.1% triton-X-100 (#85111, Thermo Scientific, United States) and incubated with 20 μg/ml RNase (#EN0531, Thermo Scientific, Lithuania) at room temperature for 30 min (Babes et al., 2018). Finally, cells were stained with PI (#sc-4252AK, Santacruz Biotechnology, United States) and immediately analyzed by a 488 nm single laser emitting device within 15 min.

### 2.5 Real-time PCR

Total RNA was isolated by using an RNA isolation kit (#740955.250, Macherey-NAGEL, Düren, Germany) according to the user’s manual. After that, isolated total mRNAs were converted in cDNAs with QuantiTect Reverse Transcription Kit (#205313, QIAGEN, Hilden, Germany). RT-PCR was performed using SYBR Green (#4309155, Thermo Fisher, Waltham, ABD) and assayed in triplicate using the iCycler RT-PCR detection system (Bio-Rad, Hercules, CA, United States). The expression levels were normalized with respect to RPL30 (Ribosomal Protein L30) gene (F: 5′-ACA​GCA​TGC​GGA​AAA​TAC​TAC-3′ R: 5′-AAA​GGA​AAA​TTT​TGC​AGG​TTT-3′) levels. Genes and their corresponding primer sequences used in this study as follows; Tumor protein 53 (TP53) (F: 5′-GCC​CAA​CAA​CAC​CAG​CTC​CT-3′ R: 5′-CCT​GGG​CAT​CCT​TGA​GTT​CC-3′) Ataxia telangiectasia mutated (ATM) (F: 5′- TGT​TCC​AGG​ACA​CGA​AGG​GAG​A - 3′ R: 5′- CAG​GGT​TCT​CAG​CAC​TAT​GGG​A-3′), Ataxia telangiectasia and Rad3 related (ATR) (F: 5′-GGA​GAT​TTC​CTG​AGC​ATG​TTC​GG-3′ R: 5′-GGC​TTC​TTT​ACT​CCA​GAC​CAA​TC-3′), Caspase7 (F: 5′-TCA​GTG​GAT​GCT​AAG​CCA​GAC​C-3′ R: 5’ –CGA​ACG​CCC​ATA​CCT​GTC​AC-3′), Caspase8 (F: 5′-GCC​ACC​CGG​CTT​CAG​AAT​GGC-3′ R: 5′-TAT​GGG​CCA​TCT​GCT​GTT​GGC​AGT-3′), baculoviral inhibitor of apoptosis repeat-containing 5 (BIRC5 or Survivin) (F: 5′-TCT​TCA​CCG​CTT​TGC​TTT​C-3′ R: 5′- CGC​ACT​TTC​TCC​GCA​GTT​TC-3′), Bcl-2-associated X protein (BAX) (F: 5′- TGC​AGA​GGA​TGA​TTG​CCG​CCG-3′ R: 5′-ACC​CAA​CCA​CCC​TGG​TGT​TGG-3′), Tyrosine-protein kinase (ABL-1) (F: 5′-TAC​CCG​ATT​GAC​CTG​TC-3′ R: 5′-CGA​TTT​CAG​CAA​ACG​ACC​CC-3′), proliferating cell nuclear antigen (PCNA) (F: 5′-CAA​GTA​ATG​TCG​ATA​AAG​AGG​AGG-3′ R: 5′-GTG​TCA​CCG​TTG​AAG​AGA​GTG​G-3′), Kinetochore-associated protein-1 (KNTC-1) (F: 5′-ATA​GTC​AAC​CCA​GAG​TGG​GCT​GT-3′ R: 5′-TTT​CAC​GTT​TTT​CGT​GCT​GCT​GCG-3′), DNA replication licensing factor (MCM2) (F: 5′-TGC​CAC​TGT​CAT​CCT​AGC​CA-3′ R: 5′-GAT​CGA​AGG​AGC​AA-3′), large tumor suprressor kinase 2 (LATS2) (F:5′-ACAAGATGGGCTTCATCCAC-3′ R: 5′-CTG​ACA​TGG​CTC​CCT​TTC​TG-3′) Yes1 associated transcriptional regulator (YAP) (F:5′-CACAGCATGTTCGAGCTCAT-3′ R:5′-GATGCTGAGCTGTGGGTGTA-3′) Salvador Family WW Domain Containing Protein 1 (SAV1) (F:5′-CCTGTGCTCCTAGTGTACCTC-3′ R:5′-GCGTAAACCTGAAGCCAGTC-3′) Neurofibromin 2 (Merlin) (F:5′-GACAGCTCTGGATATTCTGCAC-3′ R:5′-CTGCAAGGTGAGTTTGAGGG-3′). The fold changes for each sample were determined using the 2 [−Delta C(T)] method (Livak and Schmittgen, 2001).

### 2.6 Caspase activity assay

Caspase activity in HT29 and HCT116 cells were measured after treatment of cells for 72 h with each combination by using Caspase-Glo^®^ 3/7, Caspase-Glo^®^ 8, and Caspase-Glo^®^ 9 assay systems (Promega, Madison, WI) according to manufacturer’s instructions. Shortly, cells were cultured in white 96-well plates, and the following day they were treated with compounds for 72 h. Caspase levels were determined using a luminometer (Thermo Scientific- Varioskan Lux) after incubating with the kit’s reagents for 30 and 60 min.

### 2.7 Ethynyl-2′-deoxyuridine assay

EdU is a 5-ethynyl-2′-deoxyuridine analog that is absorbed into dividing cells during DNA synthesis. As a result, EdU inclusion is a marker for cell proliferation. As suggested by the manufacturer EdU Staining Proliferation Kit (iFluor 488) (Abcam, Cambridge, MA, United Kingdom; ab219801). HCT116 and HT-29 cells were seeded in 4 wells (Millicell^®^ EZ Slide, 4-well), and after 72h, cells were treated with a culture medium containing 20 μM EdU reagent. Next, cells were incubated for 2 h and were fixed with paraformaldehyde. Nuclei were stained with DAPI. All image intensities were processed using Image J software through calculating the fluorescence intensity at the DAPI - and GFP-channels and taking their ratio as a quantified read-out for EdU-positivity.

### 2.8 Statistical analysis

All data are shown as the means ± standard errors. The statistical analysis of the results was performed with an unpaired *t*-test, and graphs were drawn using GraphPad Prism 5 software. Statistical significance was determined at *p* < 0.05.

## 3 Results

### 3.1 Results

To evaluate a potential improvement in the therapeutic impact of triple combinations including CBD/CBG, curcumin, and piperine, an optimally dosed formulation was first determined based on the cell viability measurements. Next, anti-tumorigenic properties of the optimal combination were pursued in terms of inhibition of cancer cell proliferation and metastatic capacity, promoting cell death, alteration of cell cycle properties as well as the molecular pathways they engage to promote tumor suppression.

#### 3.1.1 Effect of cannabinoid compounds together with curcumin and piperine on cell survival

**FIGURE 1:**
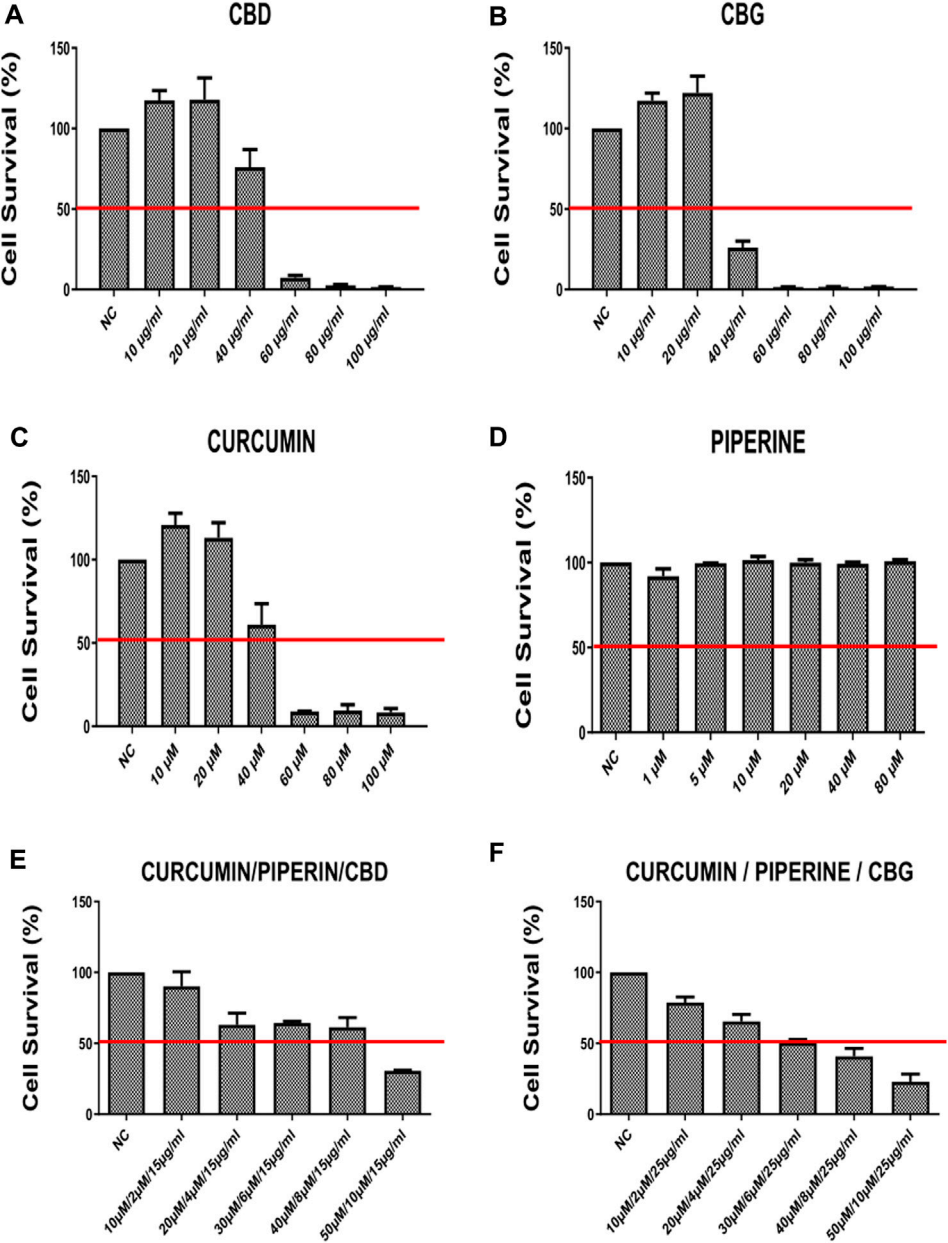
The cell survival rates (percent) of HCT-116 line at 72 h after treatment with **(A)** CBD (Cannabidiol) **(B)** CBD (Cannabigerol) **(C)** Curcumin **(D)** Piperline **(E)** combination of curcumin piperline and CBD **(F)** combination of curcumin piperline CBG. All groups were compared to their corresponding negative control.

**FIGURE 2:**
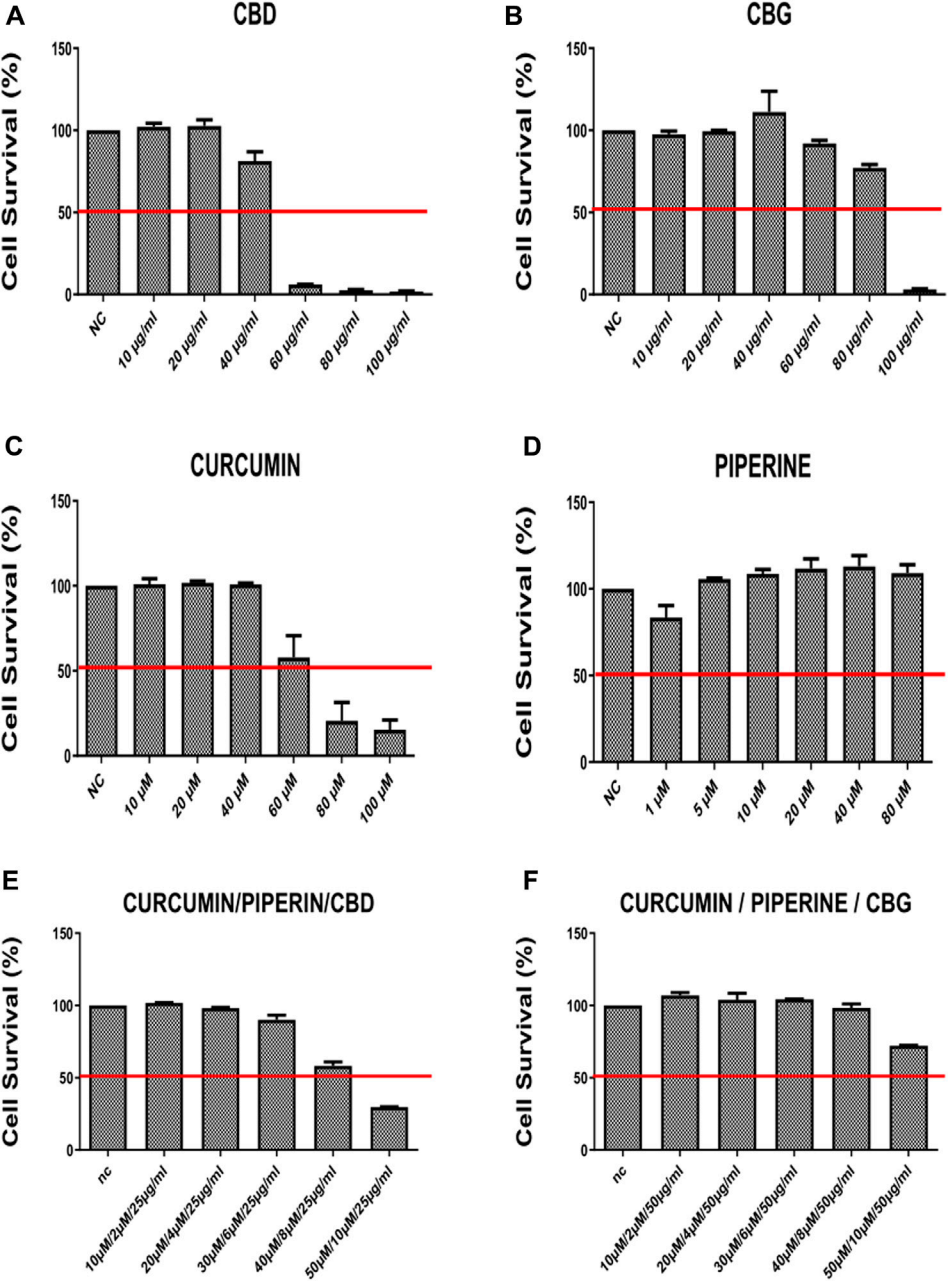
The cell survival rates (percent) of HT-29 cancer line 72 h after treatment with **(A)** CBG (Cannabidiol) **(B)** CBD (Cannabigerol) **(C)** Curcumin **(D)** Piperline **(E)** combination of curcumin piperline and CBD **(F)** combination of curcumin piperline CBG. All groups were compared to their corresponding negative control.

HT29 and HCT116 colon cancer cell lines were treated with CBD, CBG, curcumin, piperine either alone or in triple combinations at a selected range of concentrations for 72 h (Figures 1, 2). 10–100 μg/ml of CBD and CBG, 10–100 µM of curcumin, and 10–80 µM of piperine were tested in both cell lines. In all of the treatments, DMSO content relative to total volume of cell medium was kept below 0.10% v/v to avoid excessive exposure of cells to DMSO. The half maximal inhibitory concentration (IC50) values at 72 h for all treatment groups were determined in both HT29 and HCT116 cell lines using an MTS-based cytotoxicity assay (Table 1).

**TABLE 1:** IC50 values of treatment for HCT116 and HT29 cell lines.

Treatment	IC50 values of HCT116	IC50 values of HT29
CBG	94.79 (µM)	284.37 (µM)
CBD	159.2 (µM)	143.3 (µM)
Cur/Pip	50/10 (µM)	50/10 (µM)
Cur/Pip/CBD	36/7.2/47.69 (µM)	37/7.4/79.6 (µM)
Cur/Pip/CBG	30/6/79 (µM)	ND

According to our findings, curcumin, CBD and CBG alone (but not piperine) treatments reduce cell viability in a dose-dependent manner both in HCT-116 and HT-29 cells (Figures 1A–D, Figures 2A–D). Remarkably, while both cannabinoid variants were effective in HCT116 cell line (CBG being more potent) (Figures 1A, B), only CBD had a major effect on cell viability in the HT-29 cell line (Figure 2A). Interestingly, although piperine itself produces no significant change in cell viability in both cancer cell lines, its combination with CBD and CBG resulted in decreased cell viability in the HCT116 cell line compared to the negative control (Supplementary Figures S1C, E). Strikingly, although CBD (15 μg/ml) alone appeared to promote a mild increase in cell survival, combination in the triple cocktail of curcumin/piperine/CBD at the same doses promoted a cytotoxicity in the HCT116 cells displaying an additive effect based on Chou-Talalay Method (Figure 1E) (Chou, 2010). Furthermore, the doses in the triple combination of curcumin/piperine/CBG when compared to mono treatment doses displayed an antagonistic effect mildy in the HCT116 cells (Figure 1F) (Chou, 2010). On the other hand, triple combination of curcumin/piperine/CBD promoted a decrease in cell viability when combined with the non-toxic dose of CBD (25 μg/ml) and curcumin/piperine (Figures 2A, E; Supplementary Figure S2A), while curcumin/piperine/CBG did not display any cytotoxicity in the HT-29 cell line (Figures 2B, F; Supplementary Figure S2A). The obtained results indicate that the combination of CBD with either curcumin or curcumin/piperine has an additive effect in terms of decreasing cell viability in the HCT116 cell line (Figures 1A, E; Supplementary Figure S1), while combinations of cannabinoid compounds with either curcumin and curcumin/piperine showed *bona fide* antagonistic or no effect in cytotoxicity in HT-29 cells, ruling out synergism in this cell line (Figures 2E, F; Supplementary Figure S2) (Chou, 2010).

#### 3.1.2 Cannabinoid compounds, together with curcumin and piperine induce apoptosis in colon cancer cell lines

**FIGURE 3:**
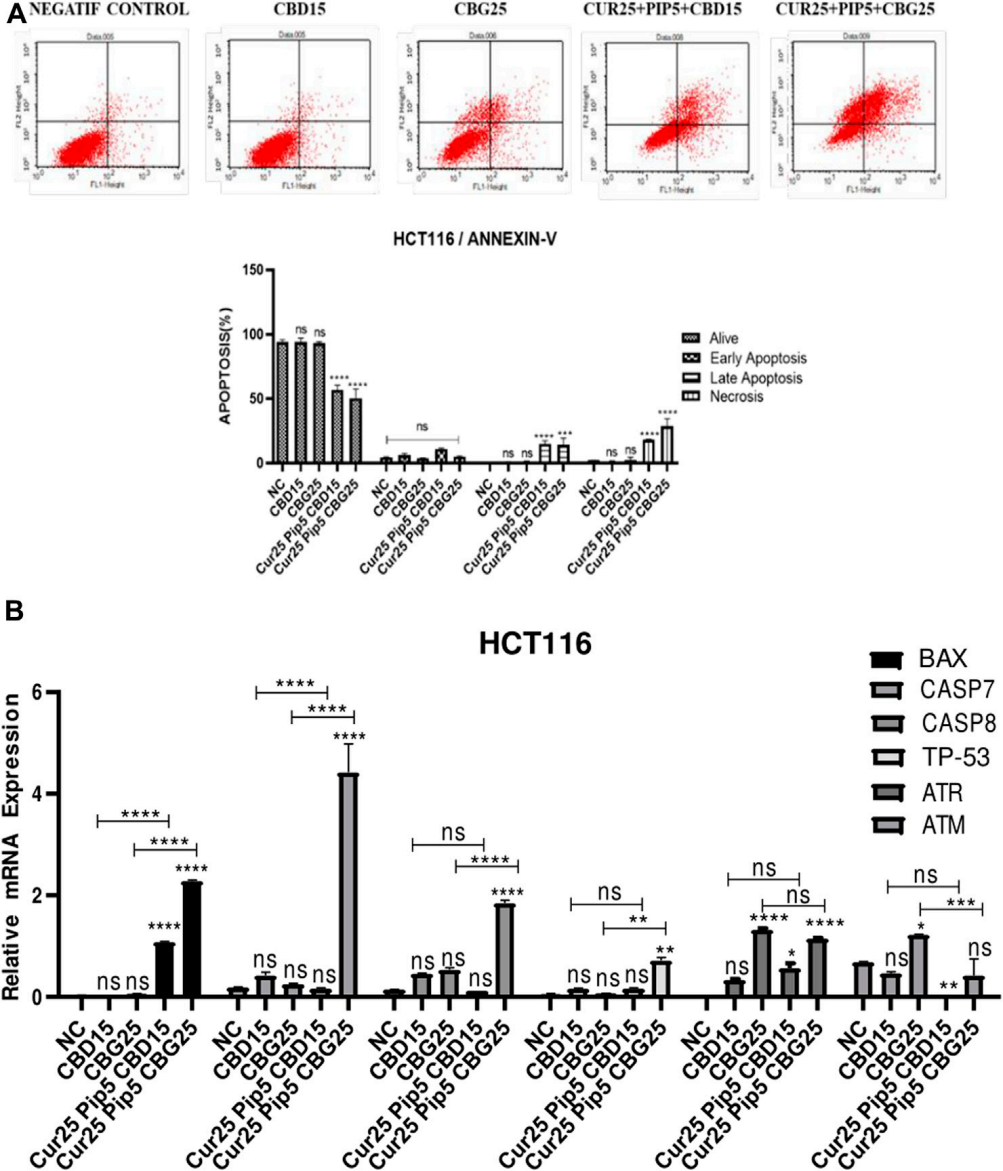
**(A)** Representative Annexin V-FITC/PI staining results for HCT116 cancer cells at 72 h quantitative analysis and their values are mean ± SD of three independent experiments **(B)** Representative graph apoptotic genes expression profiles of HCT116 cells after 72 h. (ns: non-significant, *: *p* < 0.05, **: *p* < 0.01, ***: *p* < 0.001, ****: *p* < 0.0001).

**FIGURE 4:**
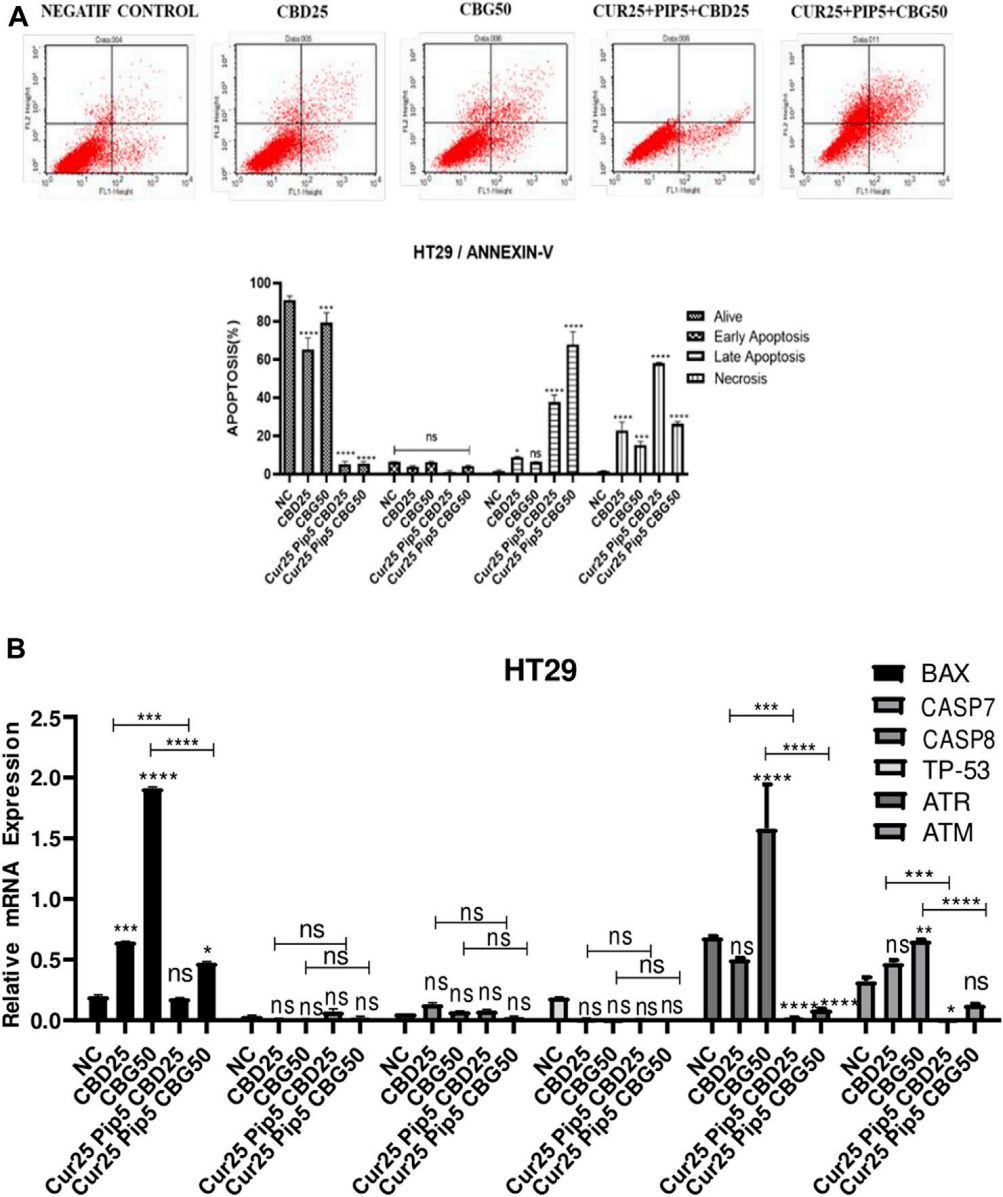
**(A)** Representative Annexin V-FITC/PI staining results for HT29 cancer cells at 72 h quantitative analysis and their values are mean ± SD of three independent experiments **(B)** Representative graph anti-apoptotic genes expression profiles of HT29 cells after 72 h. (ns: non-significant, *: *p* < 0.05, **: *p* < 0.01, ***: *p* < 0.001, ****: *p* < 0.0001).

The effect of individual or combination of the compounds on apoptosis was evaluated in HCT116 and HT29 cancer cells using the Annexin V staining protocol (Figures 3A, 4A). For each treatment group concentrations below the IC50 values were administered for 72 h. The lower left quadrant of the cytograms indicates live cells, while the right quadrant shows apoptotic cells (the lower right quadrant for early apoptotic cells and the upper right quadrant shows late apoptotic cells based on no PI inclusion). Finally, the upper left panel of the cytogram represents necrotic cells that are positive for PI. A histogram graph was drawn for further visual information (Figures 3A, 4A). Overall, treatment with CBD or CBG alone did not induce the apoptosis of HCT116 cells compared to the negative control (Figure 3A). However, application of the triple combination with either cannabinoid compounds resulted in extensive apoptotic effect (being more potent with CBD: 14.95% for late apoptosis and 11.11% for early apoptosis; CBG: 14.2% for late apoptosis and 4.88% for early apoptosis) (Figure 3A).

Mono-treatment of the HT29 cells with cannabinoid compounds increased necrosis compared to the negative control (NC by 1.68%; CBD by 22.98%; CBG by 15.35%) (Figure 4A). Furthermore, triple combination treatment with either cannabinoid compounds resulted in elevation of late apoptosis (with CBD by 37.67%; with CBG by 67.83%) and necroptosis (with CBD by 58.15%; with CBG by 26.53%), suggesting rapid initiation of programmed cell death and loss of cell membrane integrity (Figure 4A).

**FIGURE 5:**
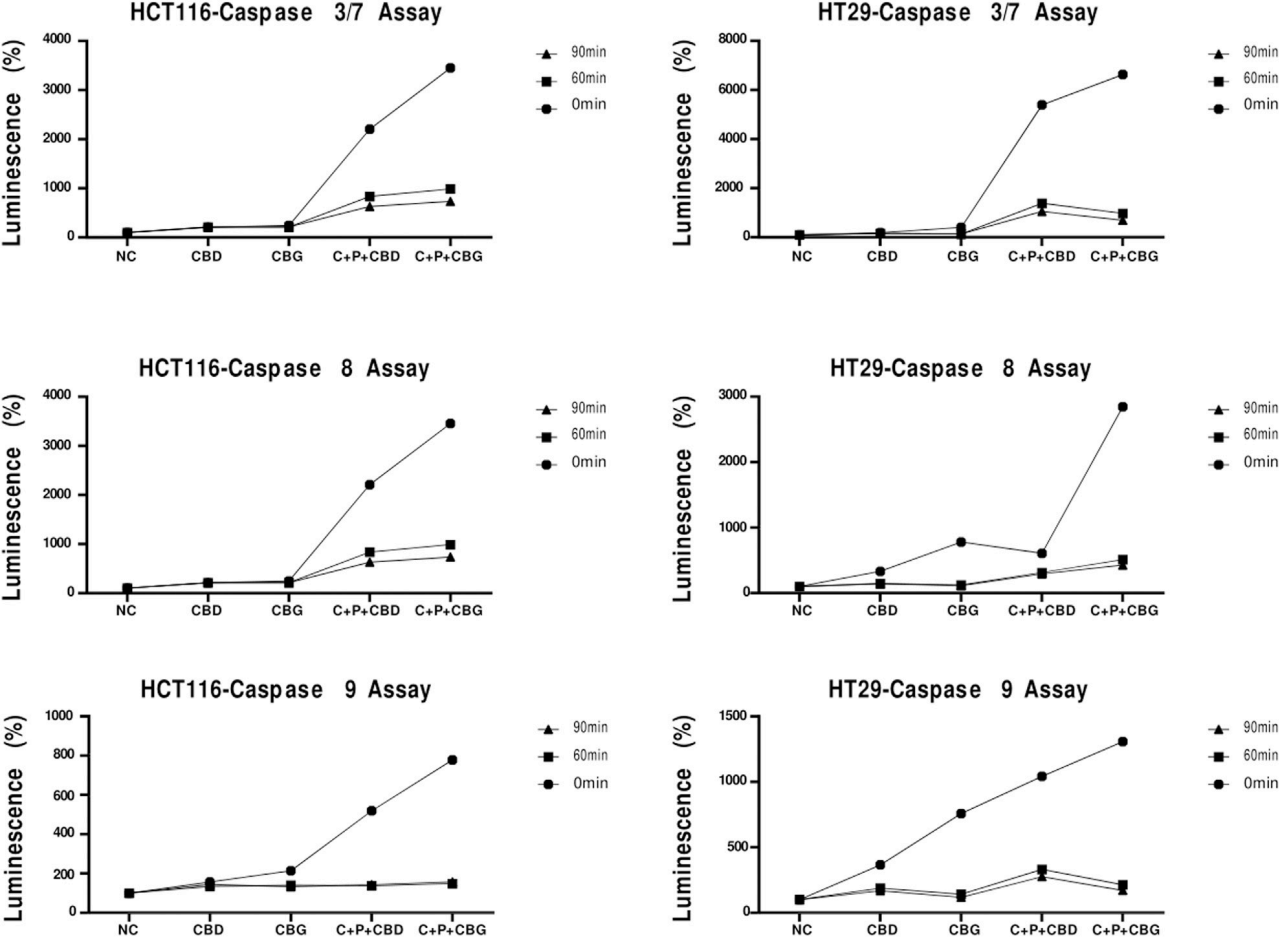
Caspase 3/7, Caspase 8 and Caspase 9 activation detected by Casepase-Glo 3/7, Caspase 8, and Caspase 9 luminescence assay for 0 min, 60 min and 90 min in HCT116 and HT29 cells that received the indicated treatments CBD (Cannabigerol), CBG (Cannabigerol), C + P + CBD (Combination of curcumin piperine CBD), C + P + CBG (combination of curcumin piperine CBG) for 72 h.

In order to further evaluate cell death in HCT116 and HT29 cells induced by the treatments at 72 h, caspase activity assay, where activities of caspase 3/7, caspase 8 and 9 were measured as a luminescence readout, was performed according to the manufacturer’s instructions. Triple combinations of both cannabinoid compounds showed elevated caspase 3/7, 8, 9 levels at 72 h, measured and displayed at 0 min in the graphic for both cell lines (Figure 5). Caspase-dependent luminescence declined over time at the post-harvest timepoints of 60 and 90 min in both cell lines (Figure 5).

To understand the exact mechanism underlying the apoptotis triggered by the compounds in this study, expression levels of hallmark genes of programed cell death were analyzed. For example, Bax (Bcl-2 associated x) is a pro-apoptotic gene and a member of the Bcl-2 gene family (Kale et al., 2018). Its expression is regulated by tumour suppressor p53 (Miyashita et al., 1994). Expression levels of both Bax (≈2.28 fold) and p53 gene (≈0.71 fold) were elevated in response to triple combination with CBG compared to untreated HCT116 cells (Figure 3B). However, there were no significant changes in the levels of p53 when these cells were treated with the triple combination containing CBD and curcumin/piperine (Figure 3B). Consistent with the changes in Bax and p53 message levels as well as the results of Annexin V assay, curcumin/piperine/CBG treatment resulted in a significant increase in caspase 7 (≈4.42 fold) and caspase 8 (≈1.84 fold) levels (Figure 3B), indicating upregulation in programmed cell death (Figure 3A). ATM (ataxia telangiectasia mutated) and ATR (ataxia telangiectasia and Rad3-related) genes encode Serine/Threonine kinases that execute a key function in DNA damage response (DDR) and cell cycle checkpoint pathways. ATR gene expression levels were significantly increased upon all treatments compared to that negative control for HCT116 cells (Figure 3B). On the other hand, while CBG mono-treatment resulted in significant increase in the levels of ATM gene, triple treatment comprising CBG restored ATM expression in untreated cells. Although CBD mono-treatment did not change the levels of ATM gene, CBD containing triple treatment lead to a dramatic decrease in the expression level of this gene (Figure 3B).

Correlating with Annexin V assay results, the mono or combination treatment schemes involving the three compounds did not alter the expression levels of caspase 7, 8, and p53 significantly in HT29 cells (Figure 4B). However, while CBG mono-treatment induced a significant increase in the expression levels of both ATR and ATM genes compared to the negative control, triple treatment with any cannabinoid compounds resulted in significant decrease in both ATR and ATM gene expression levels (Figure 4B).

#### 3.1.3 Cannabinoid compounds, together with curcumin and piperine, induce cell cycle arrest in colon cancer cell lines

**FIGURE 6:**
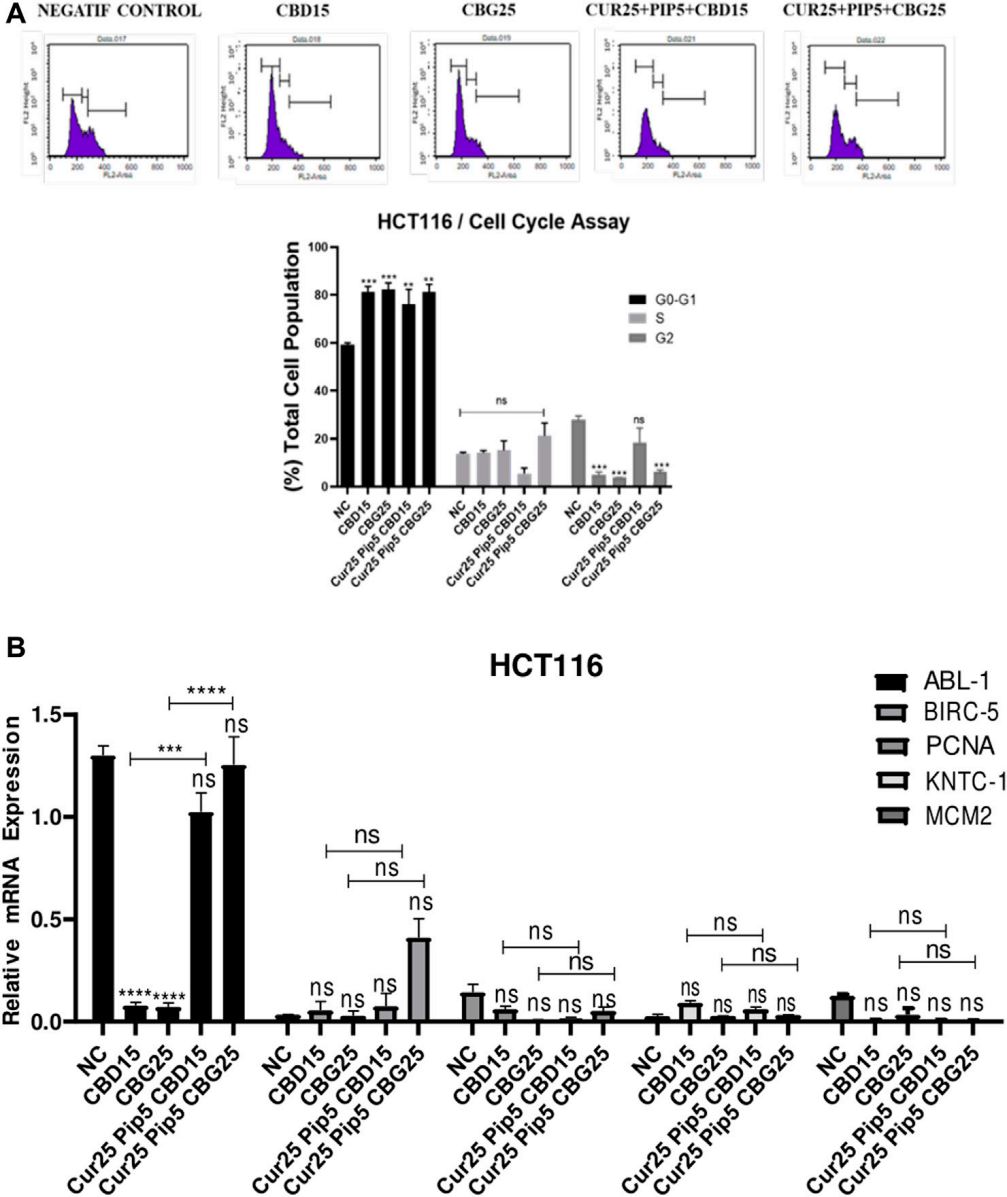
Distribution of cell cycle phases by flow cytometry for HCT116 cells **(A)** Distribution of cell cycle phases; the initial, middle, and last peaks show G0/G1, S and G2 respectively and the result of cell cycle analysis percentage of HCT116 cells in G0/G1, S and G2 phase compared with negative control. **(B)** Representative graph of cell cycle genes expression profiles of HCT116 cells after 72 h. (ns: non-significant, *: *p* < 0.05, **: *p* < 0.01, ***: *p* < 0.001, ****: *p* < 0.0001).

The observed adverse effect of the compounds of interest on cell viability can either be due to increased cell death (cytotoxic) or slowing down in the cell proliferation (cytostatic) through an arrest in the cell cycle progression. Therefore, cell cycle profiles of both HCT116 and HT29 cell lines were obtained for the same treatment conditions used in the apoptosis assays. In the mono-treatment of HCT116 cells with cannabinoid compounds an increase in the number of cells in G0-G1 phase accompanied with a decrease in the number of cells in the G2 phases compared to negative control were evident (Figure 6A). Furthermore, the triple combination, particularly the one comprising CBG, resulted in a significant increase in the G0-G1 phase population accompanied by a significant decrease in G2/M population. Non-etheless, a triple combination with CBD promoted the piling of cells in G0-G1, restoring the decrease induced in the mono-treatment in the G2/M phase (Figure 6A).

**FIGURE 7:**
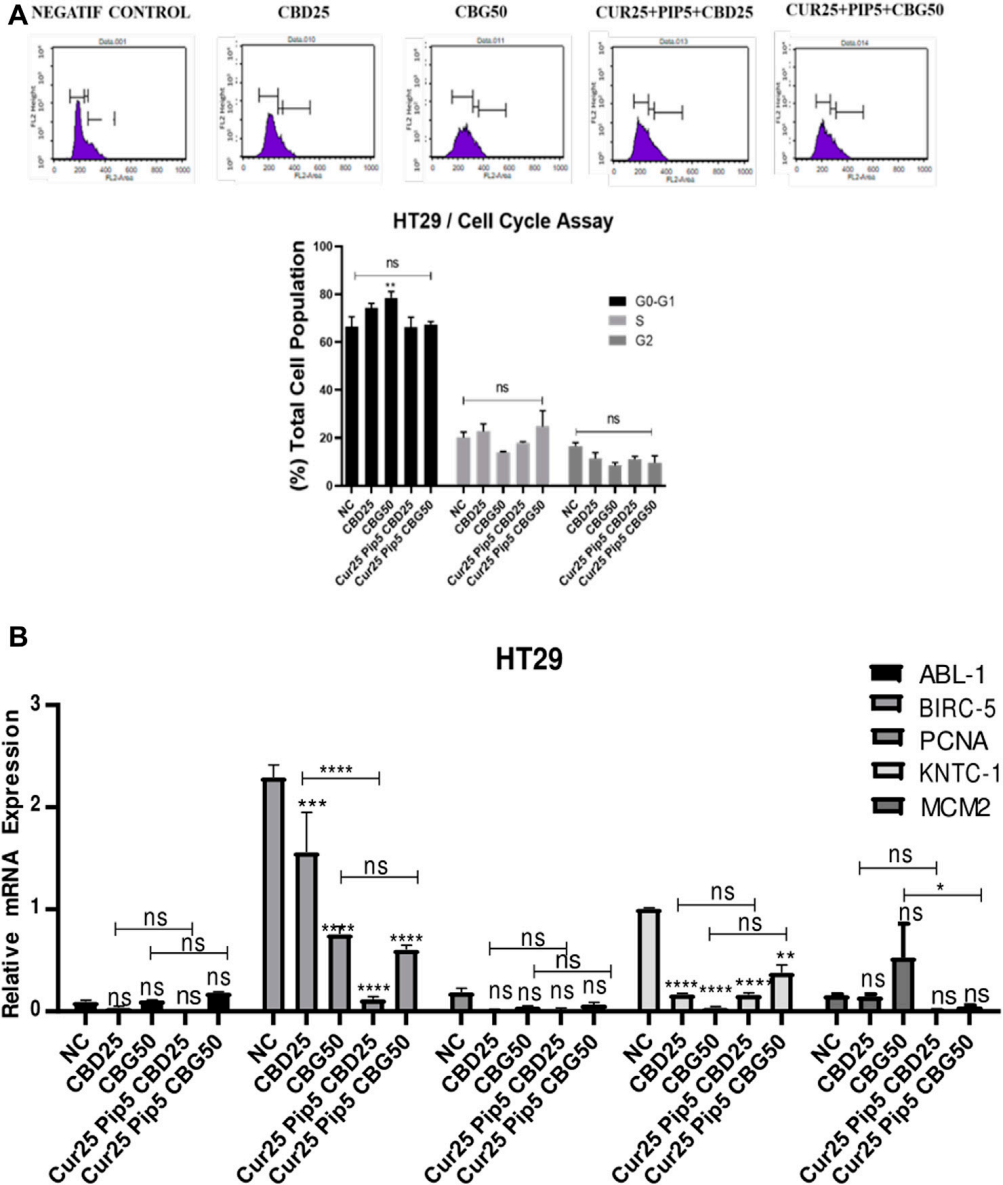
Distribution of cell cycle phases by flow cytometry for HT29 cells **(A)** Distribution of cell cycle phases; the initial, middle, and last peaks show G0/G1, S and G2 respectively and the result of cell cycle analysis percentage of HT29 cells in G0/G1, S and G2 phase compared with negative control. **(B)** Representative graph of cell cycle genes expression profiles of HT29 cells after 72 h. (ns: non-significant, *: *p* < 0.05, **: *p* < 0.01, ***: *p* < 0.001, ****: *p* < 0.0001).

In HT29 cells, except for the mild increase in G1 cells induced by CBG alone, there was a no significant alterations in the cell cycle profiles obtained with any of the treatments (Figure 7A).

Non-etheless, changes in the expression of a panel of genes, including ABL-1 (Tyrosine-protein kinase), BIRC-5 (Baculoviral IAP Repeat Containing 5), PCNA (Proliferating cell nuclear antigen), KNTC-1 (Kinetochoreassociated protein 1) and MCM2 (Minichromosome Maintenance Complex Component 2), which are also implicated in the regulation of cell cycle, were examined both in HCT116 and HT29 cell lines under identical treatment conditions (Figures 6B, 7B). In HCT116 cells, mono-treatment with cannabinoid compounds resulted in significant decrease in ABL-1 gene expression, while triple treatments restored the levels of ABL-1 (Figure 6B). No significant change was observed for BIRC-5, PCNA, KNTC-1 and MCM2 gene expression levels in HCT116 cells. However, in HT29 cells, while expression levels of ABL-1 and PCNA were not altered with any of the treatments, BIRC-5 gene expression levels were downregulated upon all treatments compared to control cells. This decreasing trend was more pronounced in the triple treatment including CBD in comparison to that seen upon CBD mono-treatment (Figure 7B). Furthermore, all the treatments resulted in a decrease in the expression levels of KNTC-1 in HT29 cells (Figure 7B).

**FIGURE 8:**
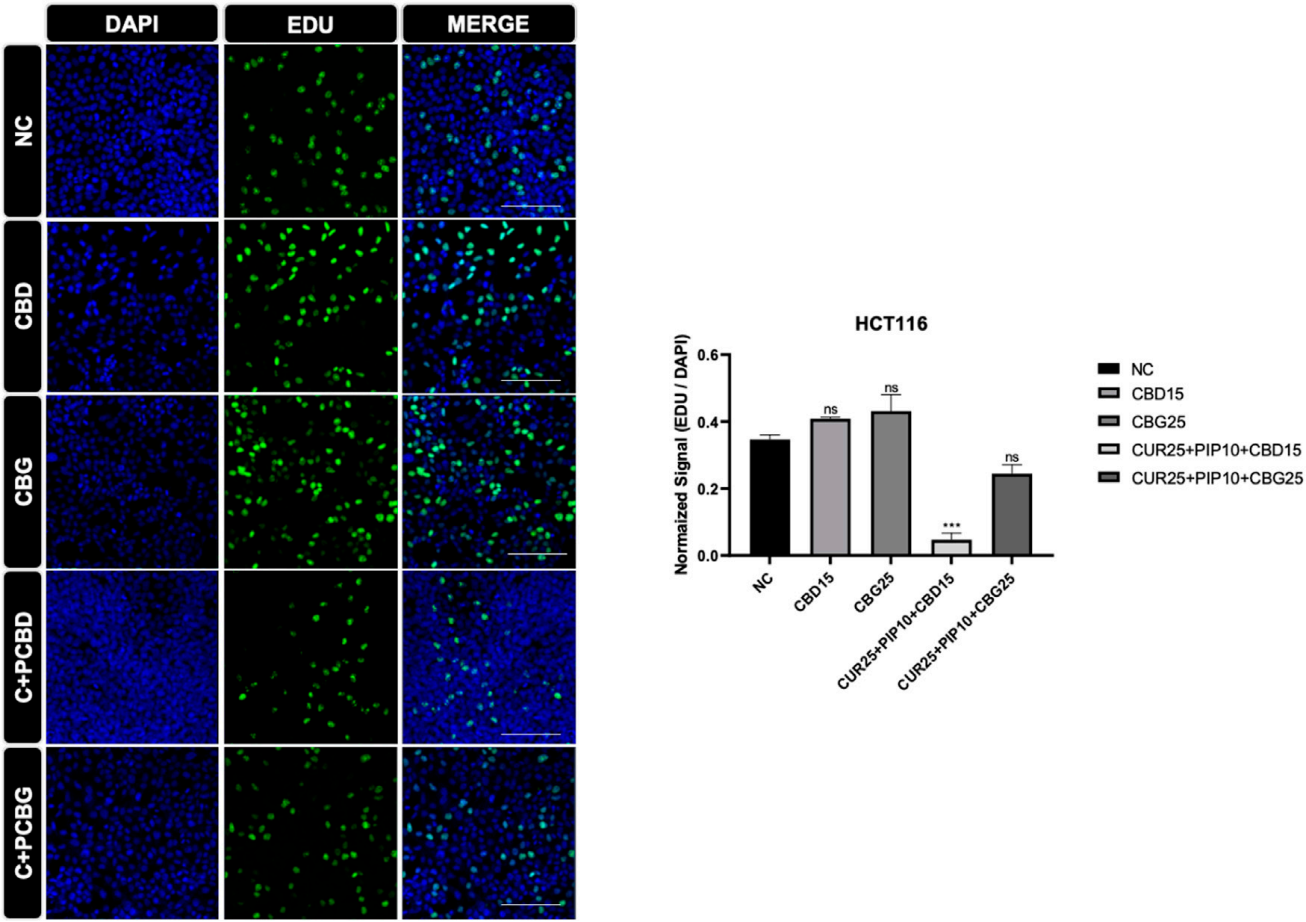
Representative results of the EDU assay are shown for HCT116 cell line. Dividing cell were labelled with EDU (green). DAPI staining was in blue in the nucleus. Histogram graph was indicated normalized signaling rate of EDU cells. (Scale bar is 100 µm) (ns: non-significant, *: *p* < 0.05, **: *p* < 0.01, ***: *p* < 0.001, ****: *p* < 0.0001).

**FIGURE 9:**
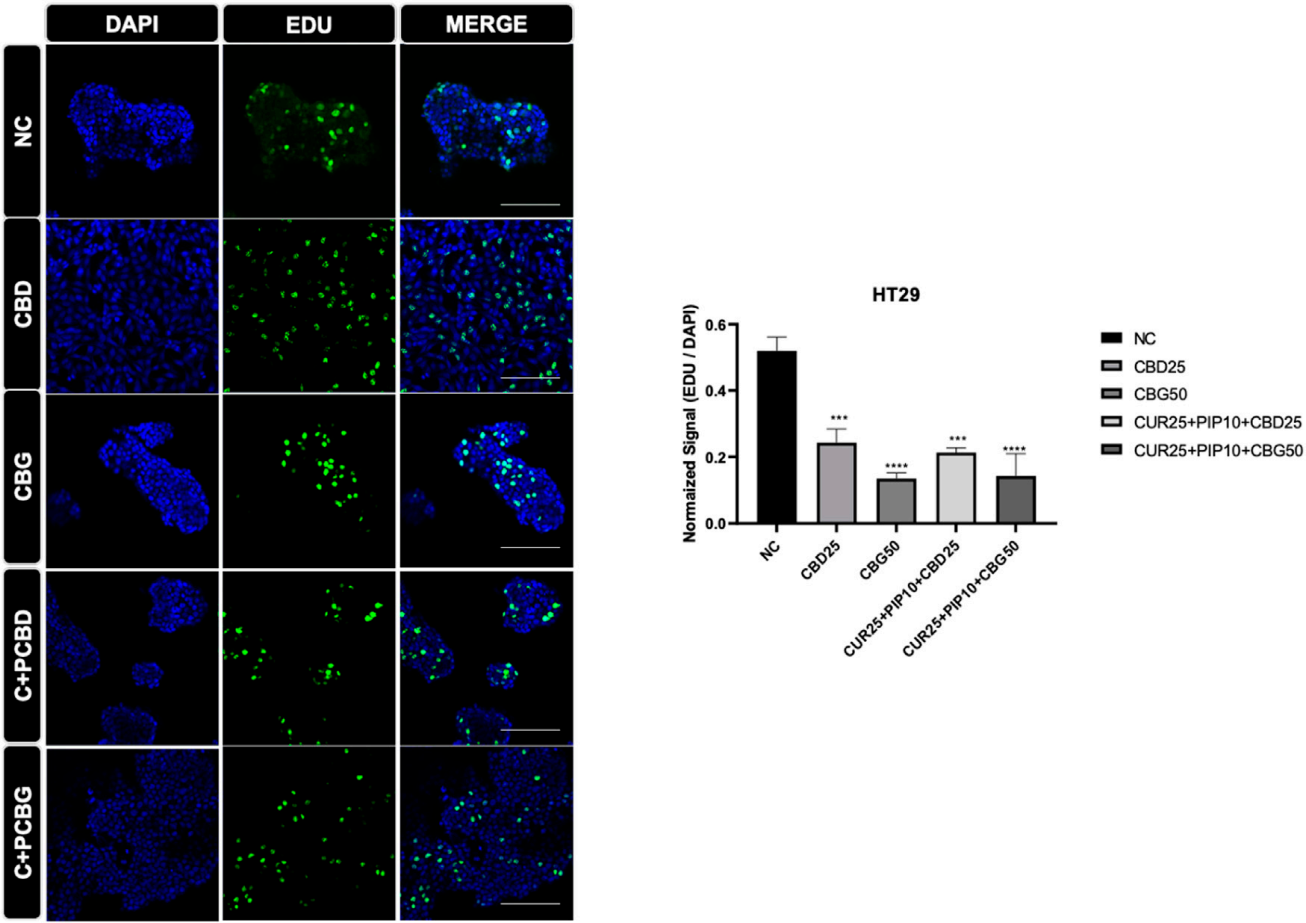
Representative results of the EDU assay are shown for HT29 cell line. Dividing cell were labelled with EDU (green). DAPI staining was in blue in the nucleus. Histogram graph was indicated normalized signaling rate of EDU cells.

The effect of cannabinoid compounds together with curcumin and piperine on the cell proliferation of HCT116 (Figure 8) and HT29 (Figure 9) cells was examined using a DNA staining-based assay. In the HCT116 cell line, cellular proliferation was significantly suppressed only upon the triple treatment comprising CBD compared to negative control (Figure 8). On the other hand, in the HT29 cells, cell proliferation was significantly decreased upon all treatment schemes (Figure 9).

#### 3.1.4 Effect of cannabinoid compounds together with curcumin and piperine on Hippo pathway

**FIGURE 10:**
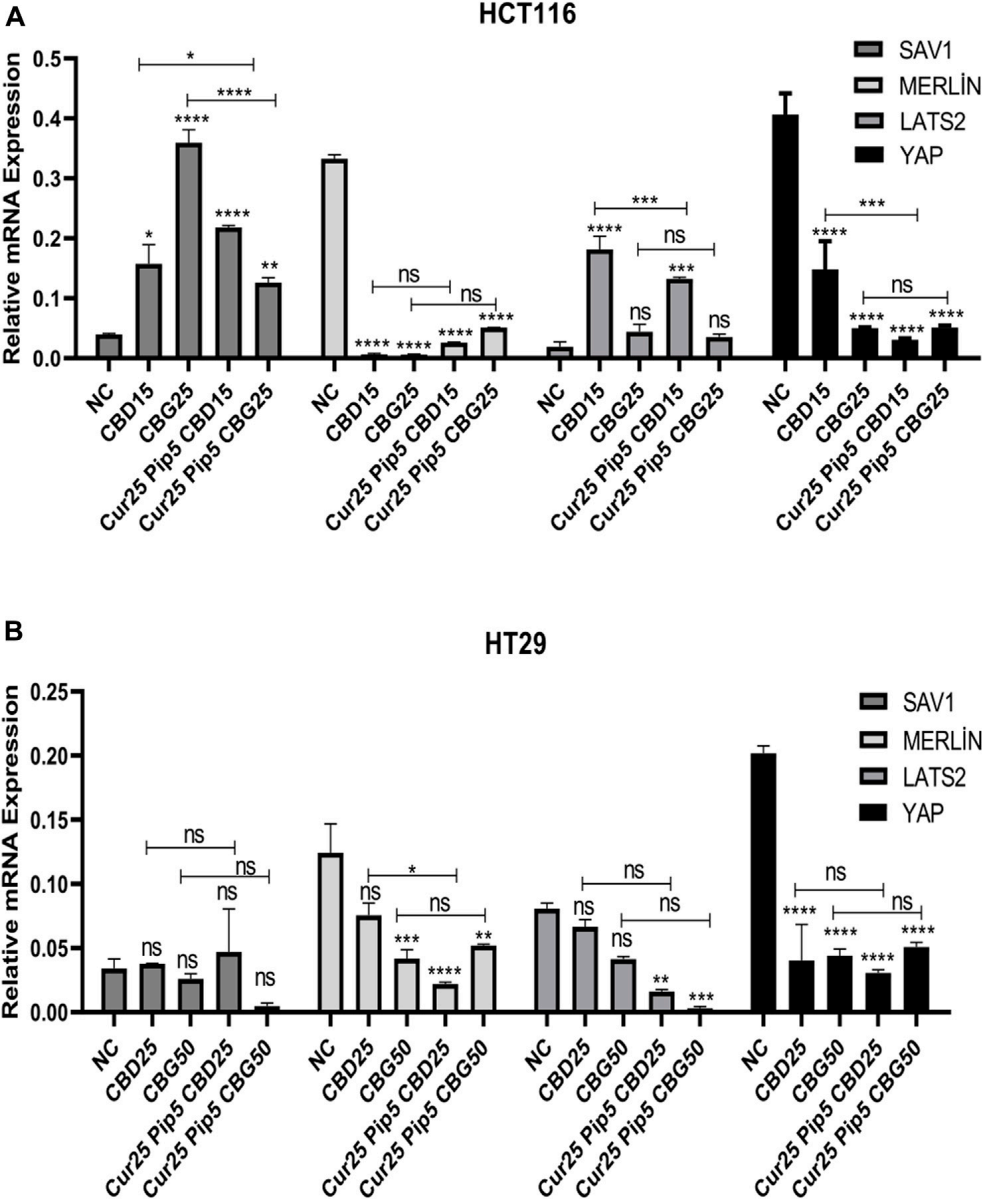
**(A)** Representative graph of Hippo pathway genes expression profiles of HCT116 cells after 72 h. **(B)** Hippo pathway genes expression profiles of HT29 cells after 72 h. (ns: non-significant, *: *p* < 0.05, **: *p* < 0.01, ***: *p* < 0.001, ****: *p* < 0.0001).

The expression of YAP, LATS2, Merlin and SAV1 genes were examined in both colon carcinoma cell lines (Figure 10). The results indicated that YAP expression levels were significantly decreased upon all treatments compared to that negative control for both HCT116 (Figure 10A) and HT29 (Figure 10B), suggesting anti-carcinogenic effects of the drugs tested depends on blocking the YAP oncogenic pathway. On the other hand, expression of the Merlin gene was decreased both in HCT116 and HT29 (only mild decrease was observed for CBD mono-treatment) cell lines for all treatments (Figures 10A, B). Furthermore, SAV1 mRNA expression was upregulated in all treatments for the HCT116 cell line (Figure 10A), while there was no significant change in the expression of SAV1 in HT29 cells (Figure 10B). Interestingly, the trend in LATS2 expression levels was in contrast in the two cell lines, in the sense that treatments comprising CBD (both mono and triple) resulted in elevation of LATS expression in HCT116 cells (Figure 10A), while the triple treatments with any of the cannabinoid compounds lead to decrease in levels of LATS2 expression in HT29 cells (Figure 10B).

Likewise, signaling downstream to suppression of oncogenic YAP pathway appears to be divergent between HCT116 and HT29 cells. While changes seen in the HCT116 background are consistent with the consequential events of the canonical YAP suppression as indicated by the significant increase in the LATS2 expression (more pronounced effect seen in mono and the triple combination consisting of CBD) in all of the treatments, a non-canonical YAP suppression accounts for the loss of tumorigenesis in the HT29 cells as implied by the reduction of LATS2 levels in the same treatment scheme.

## 4 Discussion

One of the major causes of cancer-related deaths worldwide is colon cancer, a disorder in which malignant tumors initially occur in the tissues of the colon and in the later stages of the disease, can metastasize to distal sites such as the liver, lung and ovaries. Although the standard of care for the treatment of colon carcinoma includes surgical resection, treatment with 5-FU (5-fluorouracil), and radiotherapy, all have adverse effects and unsatisfactory contributions to prognosis. Therefore, more effective and less toxic combination regimens are urgently needed in the clinic. To meet this pressing need, more and more plant-derived natural compounds targeting multiple molecular and cellular pathways in cancer cells are being investigated to bring novel therapeutic agents to the bedside (DU et al., 2013).

Among the numerous candidates tested so far, curcumin, piperine and certain types of cannabinoids performed promisingly well in colon carcinoma models as monotherapy agents. These results spurred an interest in the field to address the question whether the therapeutic potential of these agents can be boosted through combining them with other promising drug candidates or conventional chemotherapy agents. For example, Resveratrol, epigallocatechin gallate, sulforaphane and piperine are among the molecules studied for their contribution to the synergism seen in the anti-cancer behavior of their combination with curcumin (DU et al., 2013; Jin et al., 2017; Baspinar et al., 2018; Danafar et al., 2018). Meanwhile, chemo-potentiating effect of curcumin in combinations with conventional chemo-agents such as doxorubicin, docetaxel, gemcitabine, celebrex, paclitaxel, camptothecin and cisplatin is also reported in different cancer models (Kanai et al., 2011; Yan et al., 2016; Dash and Konkimalla, 2017; Abdallah et al., 2018); (Xiao et al., 2015; Li et al., 2017; Calaf et al., 2018).

However, despite the proven anti-tumor activities of curcumin, piperine and their combination, their low solubility and the poor chemical stability of the compounds in water largely limit their clinical applications. To overcome these challenges nanoparticle technology-based targeted and inducible drug delivery systems have been investigated as a prominent strategy to harness the full therapeutic potential these compounds offer (Wong et al., 2019). Polymeric nanoparticles, such as cyclodextrin nanoparticles, liposomes, copolymeric micelles, and solid lipid nanoparticles, are the most commonly applied in curcumin and piperine nanoformulations (Mahran et al., 2017).

The current study addresses the hypothesis whether a dose-wise optimally calibrated triple combination of curcumin, piperine, and cannabinoid compounds could offer a more effective therapeutic option in the treatment of colon carcinoma using established cell lines as a model system.

Here, we evaluated the cytotoxic and cytostatic effects of curcumin, piperine and cannabinoids alone and in combination in different concentration ranges on colon adenocarcinoma cell lines HCT116 and HT29. It was concluded that, although the same combination of compounds was used, each line responded differently to the treatment schemes tested. Overall, HCT116 cells displayed more sensitivity to CBD and CBG single or combination treatments compared to HT29 cells. Particularly, when HCT116 cells were treated with the triple combination including CBD induction of an additive therapeutic effect was noteworthy. Furthermore, combinations of curcumin, piperine and CBG showed more profound effect in HCT116 cells, whereas the same combinations were not effective in HT29 cells regarding cytotoxicity (Figures 1F, 2F).

One reason that could explain the differential response of the two cell lines to these agents either in single or triple form could be due to the differential expression of the target proteins that these agents interact with. For example, in their comprehensive review on cannabinoids and changes in the Endocannabinoid System (ESC) in intestinal inflammation and colorectal cancer, Cherkasova and colleagues point out that 1) in addition to CB1 and CB2, there are seven-transmembrane Gi/o-coupled receptors (GPCRs), most which are inhibitory, and respond to cannabinoids, including the most studied receptors are GPR119, GPR55, peroxisome proliferating activated receptor α (PPARα), and PPARγ, and 2) expression levels of the endocannabinoids fluctuate in response to satiety, diarrhea, emesis and inflammation, highlighting the scope of sophistication cannabinoids may elicit intracellularly (Cherkasova et al., 2021). In fact, this level of complexity becomes more advanced depending on the agonistic or antagonistic behavior of the ligand cannabinoid variant. For example, CBD-dependent induction of apoptosis via activation of p53-dependent apoptotic pathways is reported in the *in vitro* models glioblastoma multiforme (GBM), which present with high expression levels of CB1 and CB2 receptors. However, in their detailed study, Ivanov and colleagues demonstrate that as a poor ligand for CB1 and CB2 receptors, CBD-dependent signaling initiates independent of triggering of the receptors, but does engage in a downstream crosstalk with the CB1/CB2-mediated signaling in exertion of its pro-apoptotic effects (Ivanov et al., 2017).

Furthermore, differences in the genetic profile between the two cell lines, growth rate and mutations may also explain the divergent responses to these compounds. For example, HCT116 cells express wild-type forms of the BRAF and p53 genes, whereas both genes encode mutant protein forms with altered function in the HT29 cancer cell line. On the other hand, the HCT116 cell line bears mutations in the KRAS oncogene, while no mutations are reported for this gene in the HT29 cells. Although not dissected out in this study in detail, recent reports in the literature point to the importance of having a wild-type p53 status for the occurrence of a curcumin-dependent induction of apoptosis in breast and neuroblastoma cancer models (Heider et al., 2022), (Wang et al., 2022). Likewise, the detailed study by Raup-Konsavage et al. (2018) showed that among the 370 different cannabinoid compounds they tested on distinct molecular subtypes of CRC cell lines (SW480, SW620, HT-29, DLD-1, HCT-116, LS-174T, RKO), the extent of the therapeutic response was further influenced by the oncogenic mutations these cell lines carried (Raup-Konsavage et al., 2018). They also report that the cell lines with APC mutations (SW480, HT-29, DLD-1) were more sensitive to CBD than the cells mutated in the β-catenin pathway (HCT-116, LS-174T) (Raup-Konsavage et al., 2018).

Last but not least, potential direct interaction of CBD, CBG and curcumin/piperine with proteins of interest investigated in this study at the transcriptional level can be involved in eliciting differential therapeutic response in the two *in vitro* models of CRC. For example, curcumin has been shown to control the direct interaction between p53 and its binding partners in such a way that EGAP-p53 interaction becomes lost while NQO1-p53 interaction becomes promoted resulting in the profoundly increased stability of p53 protein (Patiño-Morales et al., 2020). In addition, effects of cannabinoid variant CBD in increasing transcript levels of p53 is reported in pancreatic carcinoma cells although whether this increase in p53 mRNA levels is a consequence of a direct interaction of CBD with factors that regulate p53 expression was not investigated (Luongo et al., 2020).

Another interesting study that addresses the differences between the effects of cannabinoids and curcumin as single agents and in dual combination reports an antagonistic impact of curcumin on cannabis-dependent intoxication via the Cannabinoid Receptor (Zhu et al., 2018). This antagonistic impact of curcumin has been shown in this detailed pharmacological study, where the authors base their claim on the results from binding capacity to the CB1R and other read outs such as inhibition of forskolin-stimulated cAMP accumulation, and β-arrestin2 recruitment in Chinese hamster ovary cells stably expressing human CB1R as well as different pharmacological assays (Zagzoog et al., 2022).

All these points suggest that mutational burden, possible changes in receptor expression for which the cannabinoid variant can be an agonist or antagonist, putative impact of curcumin and piperine on receptor levels, downstream crosstalk between signalizations in the cell lines used could contribute to the differential therapeutic response by HCT116 and HT29 cell lines used in this study. One powerful tool that can reduce this multifactorial rationale underlying the sensitivity versus irresponsiveness of the two cell lines involves the use of Structure Activity Relationship (SAR)-based studies prior to *in vitro* experimentation to obtain a preliminary opinion about the direct targets for these drugs.

In light of all these observations by other groups and the experimental evidence we collected in the colon carcinoma cells that are administered with these agents, anti-tumorigenic effects of cannabinoids as single agents can either be augmented or neutralized by curcumin and piperine depending on the cell line, levels of the target proteins expressed in those cells and the crosstalk between the downstream signalization these compounds trigger.

In terms of the candidate molecular mechanisms underlying anti-tumorigenic activity of the cannabinoids and their combinations with curcumin and piperine, putative involvement of YAP oncogenic pathway, which has been suggested as a biomarker for colon cancer, was investigated (Zhu et al., 2018). In this study, both mono and combination treatments promoted downregulation of YAP oncogene expression in HCT116 and HT29 cell lines, while expression levels of LATS2 and SAV1 tumor suppressor genes that are upstream players of the Hippo pathway were elevated significantly only in the HCT116 cell line. This may indicate that cannabinoid compounds, together with curcumin/piperine suppress proliferation of HCT116 cells through activating the Hippo signaling pathway, while suppression of proliferation by these compounds in the HT29 cell line was induced through a decrease in YAP expression level independently of Hippo signaling.

Since DNA synthesis is directly correlated with cell proliferation, elevated incorporation of 5-ethynyl-2′-deoxyuridine (EdU) stain enables visualization of newly synthesized DNA as a read-out for increased cell proliferation. Both types of triple cocktails of cannabinoid compounds (either with CBG or CBD) promoted a significant decrease in the rate of DNA synthesis in the HT29 cell line, whereas only the triple cocktail, including CBD but not CBG, resulted in a similarly significant decrease in DNA synthesis in HCT116 cells. Surprisingly, in the HT29 cell line all treatment regimes compared to control resulted in a significant decrease in the amount of newly synthesized DNA. It was concluded that the combination of distinct cannabinoids, such as the case of CBD in this study, with curcumin/piperine may elevate their antiproliferative effects in HCT116 cell line.

Cannabinoids have emerged as a promising novel class of anti-cancer agents that bind to cannabinoid receptors and activate multiple downstream pathways that induce suppression of cancer cell proliferation and trigger apoptosis. Their combination with curcumin/piperine resulted in a drastic induction of apoptosis based on the results of annexin V and caspase assays as well as increase in the levels of caspase 7, 8 and tumor suppressor gene Bax and TP53 in HCT116 cells. Furthermore, levels of those genes that function in the DNA repair pathways were elevated while levels of survival genes were reduced significantly upon the triple combination treatment of HCT116 cell line. On the contrary, despite the fact that mono-treatments of cannabinoids induced apoptosis and suppressed proliferation in the HT29 cell line, administration of neither cannabinoid variant together with curcumin/piperine did result in further elevation of caspase expression. Likewise, pronounced inductions in DNA repair gene levels in response to treatments of cannabinoids as single agents were lost in the triple combinations. In fact, others reported that curcumin can attenuate the intoxicating effects of *Cannabis* variants via indirect inhibition of Cannabinoid receptor 1 (CB1R) (Zagzoog et al., 2022). Therefore, we conclude that although cannabinoid compounds were effective as a single anti-cancer agents on HT29 cells, they are not suitable for combinatorial treatment with curcumin and piperine and, therefore, they have to be further assessed for their usage with other anti-cancer agents.

## 5 Conclusion

This study demonstrates that combination of curcumin, piperine and cannabinoid variants inhibit cell proliferation and induce apoptosis drastically in distinct models of colorectal cancer. Intriguingly, our findings point out that the compounds of interest, each of which are already known for their anti-tumorigenic and preventive role in colon cancer as single agents, displayed an augmented therapeutic effect in the cell lines tested. In the HT29 cell line, CBG significantly reduced cell proliferation and induced apoptosis as a monotherapy agent, whereas these anti-tumorigenic effects were overridden in the presence of curcumin/piperine. Therefore, findings from this study suggest a benefit in using cannabinoid compounds as single anti-cancer agents in the treatment of those colon carcinoma tumors that carry a genetic profile similar to that of the HT29 cell line. One major limitation of the current study was to reconcile these findings with the cannabinoid receptor 1 (CB1 receptor) and cannabinoid receptor 2 (CB2 receptor) expression profile of the cell lines used. Therefore, in future studies the link between the anti-tumorigenic effects of single cannabinoid compounds or their cocktails and the cannabinoid receptor expression should be interrogated to shed light on the differences in the responses of these cells to distinct cannabinoid-based regimens. In addition to the cannabinoid receptor status, role of other mutations in driver genes should be subject to more rigorous mechanistic studies to fully understand their role in determining the drug mechanism of action and the response to distinct treatment schemes involving cannabinoids as single agents their various combinations.

## Data Availability

The original contributions presented in the study are included in the article/Supplementary Material, further inquiries can be directed to the corresponding author.
