# Supplementary material for: Cannabinoid compounds in combination with curcumin and piperine display an anti-tumorigenic effect against colon cancer cells
Source: Front Pharmacol. 2023 Apr 26;14:1145666. doi: 10.3389/fphar.2023.1145666 (PMC10169831; doi:10.3389/fphar.2023.1145666)
Supplement: Supplementary file 1 [file Table1.docx]

**Supplementary Table 1**. Concentration of the applied concentration for HCT116 and HT29

| **Treatment** | **Concentration (HCT116)** | **Concentration(HT29)** |
| --- | --- | --- |
| CBG | 10-20-40-60-80-100 µg/ml | 10-20-40-60-80-100 µg/ml |
| CBD | 10-20-40-60-80-100 µg/ml | 10-20-40-60-80-100 µg/ml |
| Curcumin | 20-40-60-80-100 µM | 20-40-60-80-100 µM |
| Piperin | 1-5-10-20-40-80 µM | 1-5-10-20-40-80 µM |
| Cur/Pip/CBD | 10µM /2 µM /15 µg/ml  20 µM /4 µM /15 µg/ml  30 µM / 6 µM /15 µg/ml  40 µM / 8 µM /15 µg/ml  50 µM / 10 µM /15 µg/ml | 10µM / 2 µM /25 µg/ml  20 µM / 4 µM /25 µg/ml  30 µM / 6 µM /25 µg/ml  40 µM / 8 µM /25 µg/ml  50 µM / 10 µM /25 µg/ml |
| Cur/Pip/CBG | 10µM /2 µM /25 µg/ml  20 µM /4 µM/ 25 µg/ml  30 µM / 6 µM /25 µg/ml  40 µM / 8 µM /25 µg/ml  50 µM / 10 µM /25 µg/ml | 10µM /2 µM /50 µg/ml  20 µM /4 µM/ 50 µg/ml  30 µM / 6 µM /50 µg/ml  40 µM /8 µM /50µg/ml  50 µM / 10 µM /50 µg/ml |
