# Supplementary figures and images for: Cannabinoid compounds in combination with curcumin and piperine display an anti-tumorigenic effect against colon cancer cells
Source: Front Pharmacol. 2023 Apr 26;14:1145666. doi: 10.3389/fphar.2023.1145666 (PMC10169831; doi:10.3389/fphar.2023.1145666)

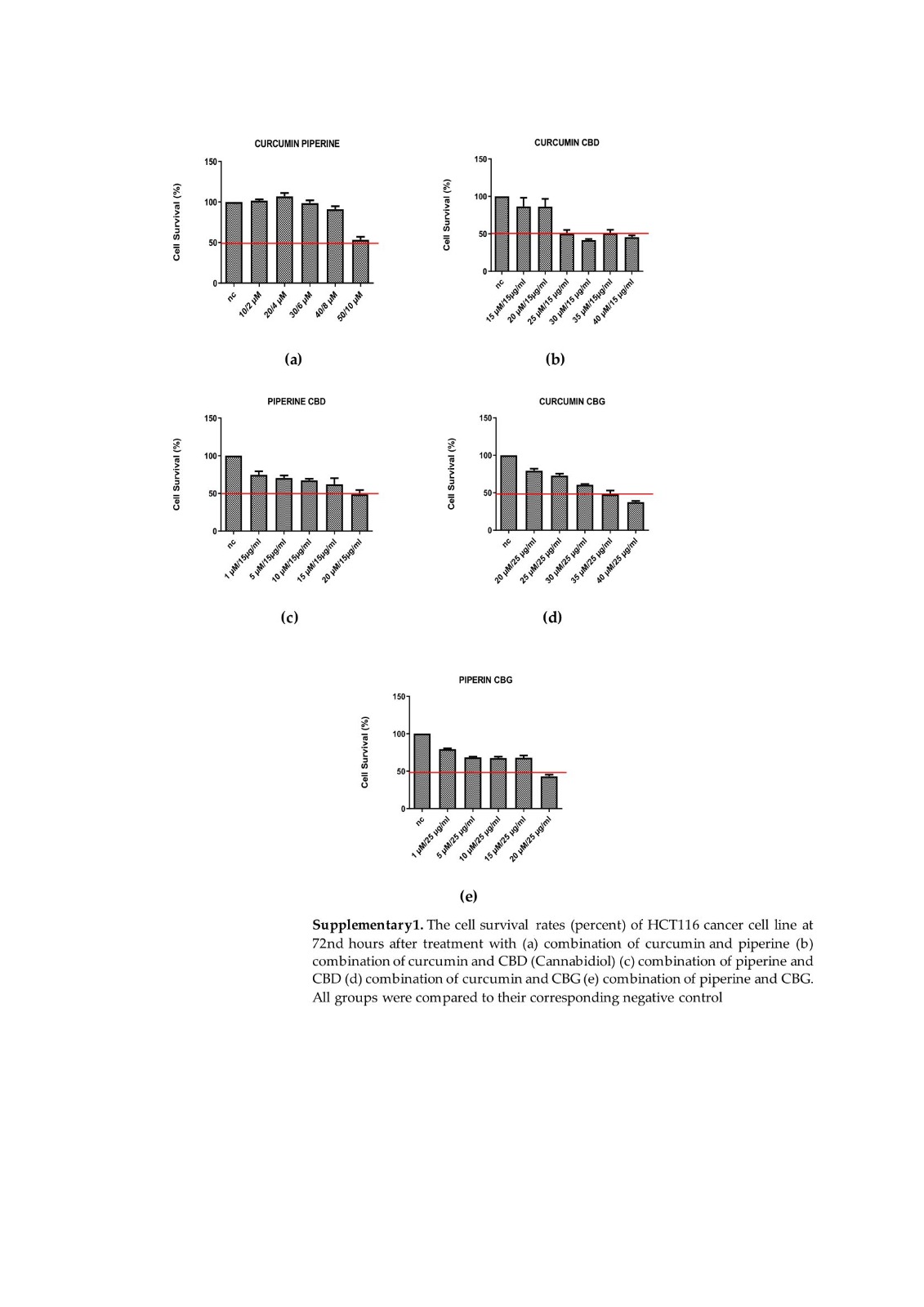

Supplement: Supplementary file 2 [file Image1.JPEG]

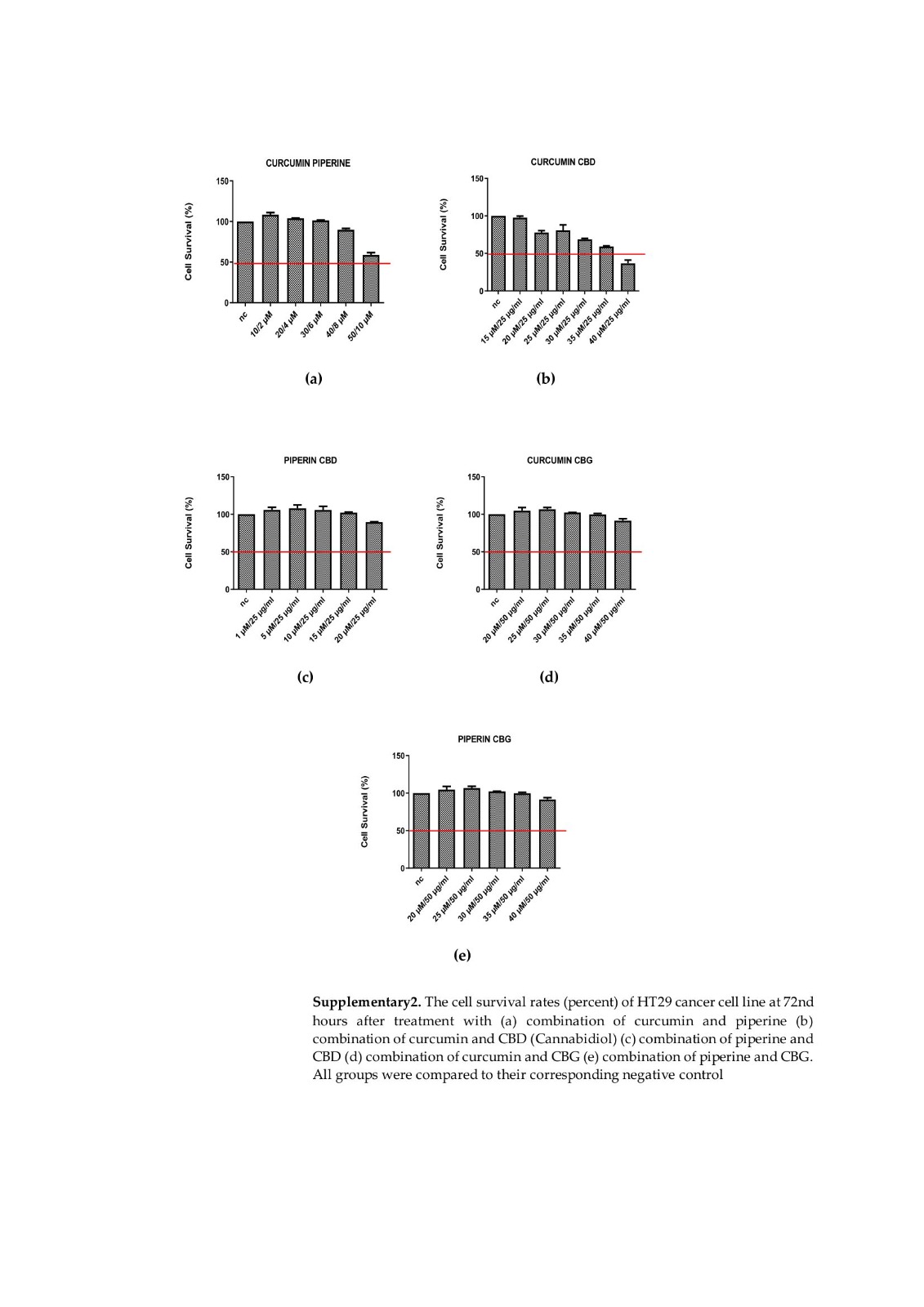

Supplement: Supplementary file 3 [file Image2.JPEG]
